# Compostability of Co-Extruded Starch/Poly(Lactic Acid) Polymeric Material Degradation in an Activated Inert Solid Medium

**DOI:** 10.3390/ma2030749

**Published:** 2009-07-08

**Authors:** Alain Copinet, Estelle Legin-Copinet, Damien Erre

**Affiliations:** 1 GRESPI (Groupe de Recherche en Sciences Pour l’Ingénieur), Esplanade Roland Garros, Pôle Technologique Henri Farman, BP1029 51686 Reims Cedex 2, France; E-Mail: damien.erre@univ-reims.fr (D.E.); 2 LMI (laboratoire de Microbiologie Industrielle), Moulin de la Housse 51686, Reims, France; E-Mail: estelle.copient@univ-reims.fr (E.L.C.)

**Keywords:** starch, poly(lactic acid), mineralisation, carbon balance, biodegradation

## Abstract

The aim of this work was to estimate the biodegradation of a co-extruded starch/poly(lactic acid) polymeric material using a vermiculite based inert solid medium which could simulate compost medium and enable us to achieve complete carbon balances. At the end of the test the mineralisation rate was compared to those obtained for co-extruded starch/poly(lactic acid) polymeric material degradation in compost. It was shown that the mineralisation rate after 45 days of degradation was similar in activated vermiculite medium to the one in compost. A protocol for both extraction and quantification of the carbon included in the different degradation by-products was proposed and the carbon balance of the polymer degradation was followed during the test with a satisfactory accuracy. As the non-degraded PLA and starch material had been retrieved during the test, the evolution of the glass transition temperature and the molecular weight of PLA could be followed. A two-step degradation mechanism was highlighted in inert solid medium, showing the fundamental role of abiotic reactions for PLA degradation in compost.

## 1. Introduction

Due to environmental concern caused by plastic accumulation, degradable polymers are of growing interest as one of the alternatives to traditional resistant petroleum-based polymers. As far as polymer compostability is concerned, its degradation is assessed by the proportion of carbon present in the polymer that is converted into carbon dioxide, disregarding the proportion of material remaining in the medium or transformed into biomass. As compost contains various chemical compounds and biological species, it is a very complex medium that makes the determination of these terms of the polymer degradation carbon balance be impossible. Recently some authors have introduced new degradation tests based on an inert and mineral solid medium [1] to deal with the problems and restrictions inherent to degradation tests in compost. These kinds of supports were used to reach enhanced repeatability and to avoid priming effects [2] or were used for quantitative and qualitative recovering of potential toxic by-products released during the degradation process. Although this subject is approximately ten year old, few articles report the utilisation of inert solid medium for carbon balance determination in solid medium. Some authors proposed a first carbon balance for the degradation of a multi-phase material composed of starch, poly(ε-caprolactone), Estane 54351 (thermoplastic poly(ε-caprolactone)-type polyurethane (BF Goodrich, USA) and small amount of plasticizers [3].

In a previous study [4], we have developed a strategy of a model compost based on inert solid medium composed of vermiculite that allows an accurate determination of the complete carbon balance. In this work, we used starch as a “model biodegradable polymer”.

In the present paper, this strategy is applied to the degradation of a new co-extruded starch/poly(lactic acid) polymeric material composed of 19.4% of lactic acid and 80.6% of starch. A particular attention was paid to the evolution of some physico-chemical properties of the non degraded PLA material [5].

## 2. Experimental Section

### 2.1. Extruded Material

Poly(lactic acid) was first plasticized with 10% by weight of polyethyleneglycol (Part **A**). At the same time, a starch-based formulation was prepared containing 65% starch and 35% glycerol (Part **B**). Then these two components were co-extruded using two single screw extruders with a die of 2 mm thickness (SCAMEX) to form an **A**/**B**/**A**-type multilayer film. The various parameter values for Parts **A** and **B** listed in Table 1 were measured after delamination of an entire film. Percentage moisture rate was measured after a one week storage for at 23 °C at a relative humidity of 50% on a crushed sample using the Karl Fischer method (NF VO3-625). Elemental analyses were carried out on crushed samples that had been dried at 105 °C for 24 hours.

**Table 1:** Main characteristics of the **A** (PLA) and **B** (starch) parts of the co-extruded film **A/B/A**.

	Proportion (in weight)	Thickness (mm)	Humidity percentage	Elementary analysis (%)		
				Carbon	Hydrogen	Nitrogen
**A (PLA)**	19.4 ± 0.3	0.66 ± 0.05	5.4 ± 0.2	48.96 ± 0.10	5.00 ± 0.06	0.20 ± 0.002
**B (starch)**	80.6 ± 0.5	0.87 ± 0.06	10.5 ± 0.3	38.57 ± 0.15	6.74 ± 0.04	0.10 ± 0.01

### 2.2. Equipment

**Figure 1:**
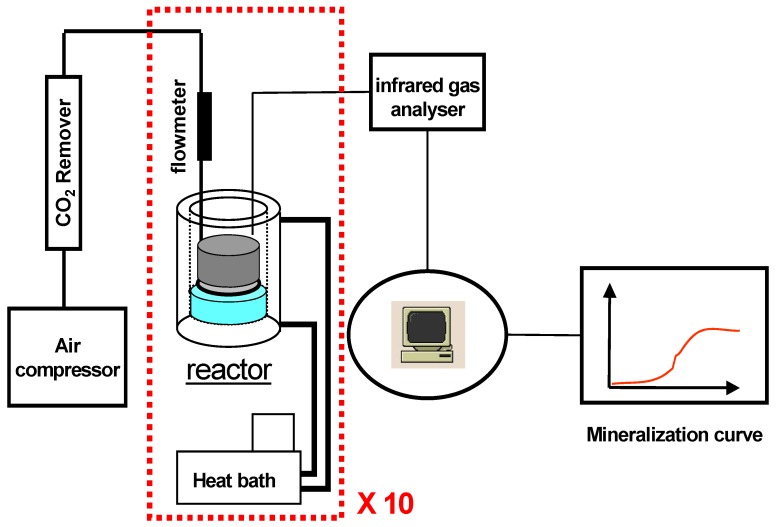
Schematic representation of the biodegradation test bench.

Our automated degradation test bench (Figure 1) consists in ten 2-liter glass bioreactors (CMF 100, Chemap, Switzerland). The temperature was controlled by double-wall water flow. The airflow in the reactors was adjusted to 0.56 L·min^-1^ by a rotary ball meter (KAG 1398, Brooks Instruments, Veenendaal, The Netherlands) and was CO_2_-free thanks to an online CO_2_ remover (CO_2_RP140, Domnick Hunter, USA). This allowed to maintain of aerobic conditions and permitted the assessment of the percentage of CO_2_ produced (scale 0-2%). Gas flow was measured by a mass flow-meter (5850 Tr, Brooks Instruments, Veenendaal, The Netherlands). CO_2_ emissions from the bioreactors were measured automatically every other hour by an infrared gas analyser (Binos 120-M, Rosemount, France). An automated acquisition system (Allen-Bradley, Millwaukee, WI, USA) was used to transfer data to a computer (Rsview 32, Rockwell Software, Velizy-Villacoublay, France). The final quantity of CO_2_ produced was calculated by integrating the CO_2_ percentage and the gas flow during the sampling time (12 minutes) [6].

### 2.3. Degradation parameters

The studies were carried out with about 2 cm x 2 cm pieces of film. The degradation test begins by placing 18.5 g of film, containing approximately 12 g of carbon, in the reactor.

#### 2.3.1. Compost test set-up

Compost (560 g) was placed in the reactor on a home made stainless steel stand, to keep the compost above 150 mL of distilled water. Air was bubbled through the distilled water to maintain water content of at least 70%/80% by weight.

Before the degradation test began, the compost was kept in bioreactors at 58 °C for about 4-5 days because of the CO_2_ production peak caused by the biological activity renewal (Figure 1). This peak could hide the CO_2_ produced by the degradation of the substrate in the first days of the test. The test began by adding the substrate in compost. All the experiments were performed in triplicate and monitored at 58 °C [7].

#### 2.3.2. Inert solid medium tests set-up

##### 2.3.2.1. Inoculum

For inert solid medium tests, reactors had been inoculated with a compost extract in order to supply the medium with micro-organisms. This extract had been prepared by adding 15 g of compost in 150 mL of a Ringer solution (Biokar Diagnostics, Bauvais, France). After a one hour homogenization by magnetic stirring, the solution was slipped through a 0.125 cm sieve and the filtrate was centrifuged at 10,000 rpm for 20 min (MR 1822, JOUAN, Saint Herblain, France). The supernatant was discarded and the solid residue was then dispersed in 150 ml of Ringer solution to form the inoculum solution which contained less than 100 mg carbon L^-1^.

##### 2.3.2.2. Non activated medium tests

One bioreactor is contained 180 g of vermiculite, 410 mL of mineral solution (Table 2), and 15 mL of compost extract. The mineral solution was buffered at pH = 7.2. This medium was standing on a home-made stainless steel stand to keep it above 250 mL of distilled water. The inlet air flow bubbles through this distilled water to maintain water content of at least 70% by weight. The degradation test began by adding 18.5 g of co-extruded starch/poly(lactic acid), containing 7.1 g of carbon, in the reactor. Every reactor content was manually agitated every other day and the test temperature was also of 58 °C. The degradation test lasted 45 days long. Experiments were conducted in triplicate [8].

Although we have a CO_2_ remover before each reactor airflow enters, an empty “blank” reactor was carried out to determine the quantity of carbon dioxide present in the inlet air flow.

**Table 2:** Composition of the mineral solution for inert solid medium tests.

Mineral salts	Composition for 5 L (g)
CaCl_2_,2H_2_O	0.6500
Na_2_HPO_4_,2H_2_O	34.8500
KH_2_PO_4_	18.7500
(NH_4_)_2_SO_4_	20.0000
MgSO_4_,7H_2_O	1.0000
FeSO_4_,7H_2_O	0.0135
MnSO_4_,7H_2_O	0.0050
ZnSO_4_,7H_2_O	0.0050
H_3_BO_3_	0.0050
KI	0.0050
(NH_4_)_6_Mo_7_O_2_,4H_2_O	0.0050

##### 2.3.2.3. Activated medium tests

This activated vermiculite medium was supposed to be suitable to simulate starch and PLA degradation in compost. Actually, micro-organisms in compost already have the enzymatic package which is necessary for the degradation of a carbon source like starch and PLA. On the contrary, micro-organisms of non-activated vermiculite, (which come from the compost extract) are in a very low quantity and haven’t produced enzymes for starch degradation yet. So with activated vermiculite tests and before the degradation takes place, a first growing phase with a high enzymes synthesis activity may have occurred at 58 °C and 80% HR recommended by the standard ISO 14855. As a consequence, mineralization would occur more slowly [9].

Before the biodegradation test took place, the medium (vermiculite) was supplemented with 15 mL of inoculum and also with a mixture containing starch, cellulose, urea and a nutrient both in various amounts. The compositions (g) of every component are given in Table 3.

During the degradation of those substrates (5 days), we daily had observed the mineralization curves to determine when we could begin biodegradation tests by adding starch in the medium. All the experiments were conducted in triplicate. The degradation test began by adding 18.5 g of co-extruded starch/poly(lactic acid).

**Table 3:** Composition of the activation dose added to the vermiculite medium.

Compound	Weight (g)
Wheat Starch	1.5
Cellulose	1.5
Nutrient Broth	1.0
Urea	0.4

### 2.4. Sampling

Five samples were taken from each reactor. The first sample was taken at the end of activation phase before adding PLA. Sample weight was approximately 15 g and the total weight of all samples for one reactor did not exceed 10% of the total weight of the medium. Samples were stored at –20 °C before being analysed to prevent further evolution.

### 2.5. Samples analysis

**Figure 2:**
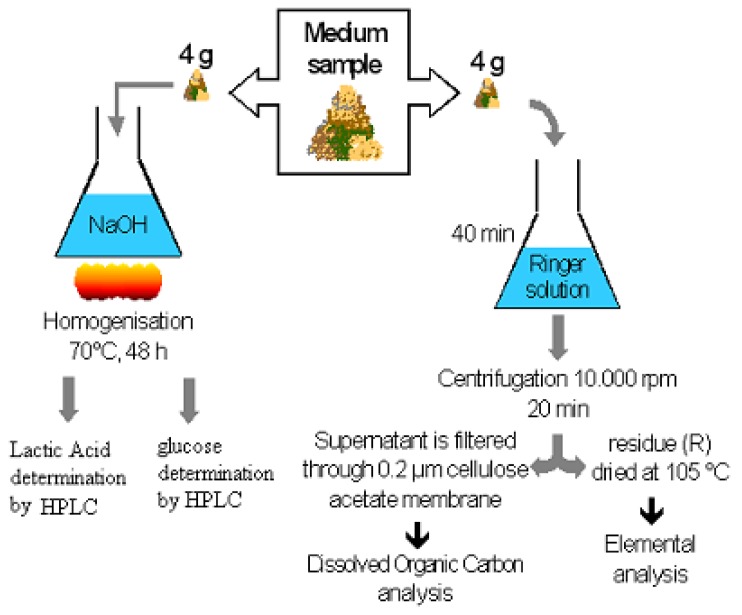
Protocol for by-product extraction and carbon quantification.

The protocol for degradation by-products extraction and carbon quantification is summarised in Figure 2. More precisely, starting from a frozen sample, 8 grams divided in two samples of 4 grams each were dried at 105 °C for dry weight determination. Then, the first four grams were homogenized in 30 mL of Ringer solution (Table 4) for 40 minutes. After centrifugation at 10,000 rpm for 20 minutes, the supernatant was filtered and a part was submitted to dissolved organic carbon analysis (DOC). The residue R was dried in an oven for two days. After it was weighed for dry weight determination, it was grounded and submitted to elemental analysis (TOC 5000 Shimadzu). The four other grams were placed in a known volume of NaOH solution (0.025 M) which was homogenized for 48 h in an oven at 70 °C. As a result, ester bonds of the PLA and the starch remaining in the medium sample were totally broken, releasing lactic acid and glucose in the solution. This solution was filtered and starch hydrolysate sugar rates measured by HPLC (Model 8880, TSP, Les Ulis, France) using the same procedure [9]. The lactic acid was also analysed by HPLC using the same apparatus but using an Aminex HPX 87H column, 300*7.8 mm (Bio-Rad, Ivry/Seine, France) maintained at 35 °C. The mobile phase (0.02 mol/L sulfuric acid) was degassed by slipping through a 0.2 μm pore size filter. A flow rate of 0.6 mL/min was maintained at 900 psi pressure. Lactic acid was measured using a UV detector (TSP 8880, Les Ulis, France). The injection volume of sample was 20 μL. Prior to injection, the samples were filtered through 0.22 μm pore size filters (Millipore Corporation, USA). Three filtered solutions corresponding to three replicated experiments were combined. A calibration curve was established beforehand using different concentrations of lactic acid (Sigma Chemical Co., Saint Louis, MI, USA).

**Table 4:** Composition of Ringer’s solution.

Mineral salts	Composition for 1 L (g)
NaCl	9
KCl	0.42
CaCl	0.48
NaHCO_3_	0.2

### 2.6. Carbon balance

The carbon balance of PLA degradation can be established as follows:

(1) C_polymer_ = Cb + Cg + Cs + Cnd

where Cg = C(CO2 out-going air) - C(CO2in-going air).

All the degraded carbon corresponds to:

(2) Cd = Cb + Cg + Cs

where the polymer carbon C_polymer_ is converted by micro-organisms into biomass carbon C_b_, carbon dioxide C_g_ and water soluble by-products C_s_. The polymer carbon which is not yet degraded is represented by C_nd_.

C_g_ was given by the gas infrared analyser of the automated degradation test bench. Water soluble degradation by-products were quantified by DOC analysis of the supernatant. The remaining polymer was determined by electrophoresis analysis after complete hydrolysis of non degraded PLA in the NaOH solution. The lactic acid concentration was equal to that of original constitutive units of non-degraded PLA. We have considered that one unit weighed 72 g/mol and the carbon content of PLA was 48.96%. To confirm our analyze and to realize carbon balance, the elemental analysis was made and the residue R quantified the amount of carbon contained in the biomass and also in the non degraded PLA (C_R_) as shown in Figure 2. Thus, the quantity of polymer carbon converted to biomass was equal to C_R_ minus C_nd_ (C_R_ = Cnd+Cb, using elemental analysis).

### 2.7. Extraction and analysis of remaining PLA material

For every sample, we performed an extraction of the non-degraded PLA material with the aim to follow the evolution of both molecular weight and glass transition temperature. We proceeded as follows: 4 g of frozen sample were homogenized in 30 mL of Ringer solution during 40 min to eliminate soluble by-products (the homogeneization time is kept sufficiently short to avoid chain scissions by hydrolysis). The solution was filtered with an cellulose acetate membrane (porosity: 0.2 µm) and the solid phase was dried at 60 °C until the weight remained stable (this drying step was performed at low temperature to avoid any thermal degradation). Then the residue was placed in 30 mL of chloroform for 10 minutes to dissolve PLA remaining in the medium sample. After a centrifugation step at 10,000 rpm for 20 minutes to remove the bulk of mineral medium, the supernatant was placed in a syringe and filtered (0.2 µm, Millipore, USA) for solvent casting in a Petri dish. After solvent evaporation, the recovered PLA was used for Size Exclusion chromatography and Scanning Calorimetry determinations.

#### 2.7.1. Physico-chemical analysis of remaining PLA material

##### 2.7.1.1. Molecular Weight Distribution of PLA Films

Gel permeation chromatography (GPC) testing was performed on samples of PLA films using a Thermo Separate products model 300 (les Ulis, France) high-performance liquid chromatograph. Tetrahydrofuran (THF) was pumped at 1ml/min and an injection volume of 200 µL were used for HPSEC analysis to determinate Mn (Number average molar mass) and Mw (Weight average molar mass). A PLA sample of 0.1 g was dissolved in 5 mL of THF. A refractive index detector (Shodex RI 71) was interfaced to PIII personal computer. A PL Caliber logiciel and interface PL-DCU ( Polymer Laboratories) enabled chromatogramms to be numerically recorded. The Plgel 5µm MIXTE-C column (Polymer Laboratories) was used. A molecular weight calibration curve was built based one ten narrow-molecular weight-distribution polystyrene standards (Easical), with M_w_ peaks ranging from 580 to 7,500,000 g·mol^-1^.

##### 2.7.1.2. Differential scanning calorimetry analysis

DSC (Differential scanning calorimetry) analysis were performed on a TA-Instruments M-DSC apparatus (USA) with approximately 12 mg of PLA. First, the sample was equilibrated at 0 °C for 3 minutes, heated (10 °C/min) and stabilized at 180 °C for 2 minutes. Then the sample was cooled down to 0 °C at 20 °C/min and maintained at this temperature for 5 minutes. The final step was a heating ramp at 10 °C/min until 200 °C and a return to room temperature. The glass transition (Tg) temperature was determined in the second heating step.

## 3. Results and Discussion

### 3.1. Mineralization rate

**Figure 3:**
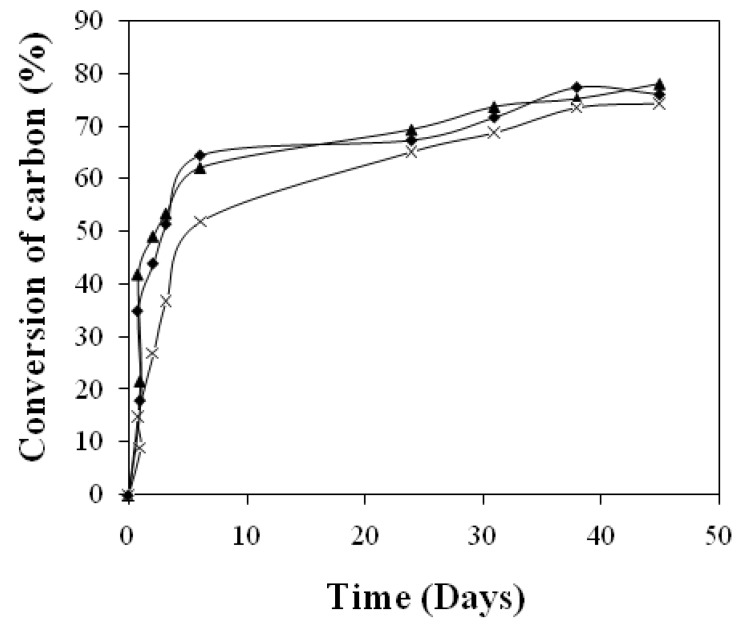
Mineralization curve for co-extruded film degradation during the tests inthe activated inert solid medium (♦), no activated inert solid medium (x) and medium compost (▲).

As it can see on the Figure 3, co-extruded starch/poly(lactic acid) polymeric material degradation is faster in activated vermiculite than in non-activated vermiculite, whatever the inoculum amount is. Actually, in activated vermiculite micro-organisms have created the enzymatic package which is necessary to the degradation of a carbon source like starch. On the contrary, micro-organisms of non-activated vermiculite, (which come from the compost extract) are in a very low quantity and have not produced enzymes for starch degradation yet. In the first hours of the non-activated vermiculite tests and before the degradation takes place, a first growing phase with a high enzymes synthesis activity may have occured. As a consequence, mineralization would occur more slowly.

The activated vermiculite medium gives a mineralization curve which is very close to the compost one. During the linear part of the mineralization curves, the conversion rate of co-extruded starch/poly(lactic acid) polymeric material in carbon dioxide is 8.3 mmol/h in compost and 8.51 mmol/h in activated vermiculite medium versus 6.45 mmol/h with no activated vermiculite. The difference between the two final mineralization percentages is about 2%. According to those results, we have considered that this particular activated inert solid medium was suitable for the degradation compost test simulation of co-extruded starch/poly(lactic acid) polymeric material. Furthermore, we decided to compare degradation of co-extruded starch/poly(lactic acid) polymeric material using inert solid activated and no activated media by performing complete carbon balances and physico-chimical properties of residual polymer.

### 3.2. Carbon balance

#### 3.2.1. Activated vermiculite medium

**Figure 4:**
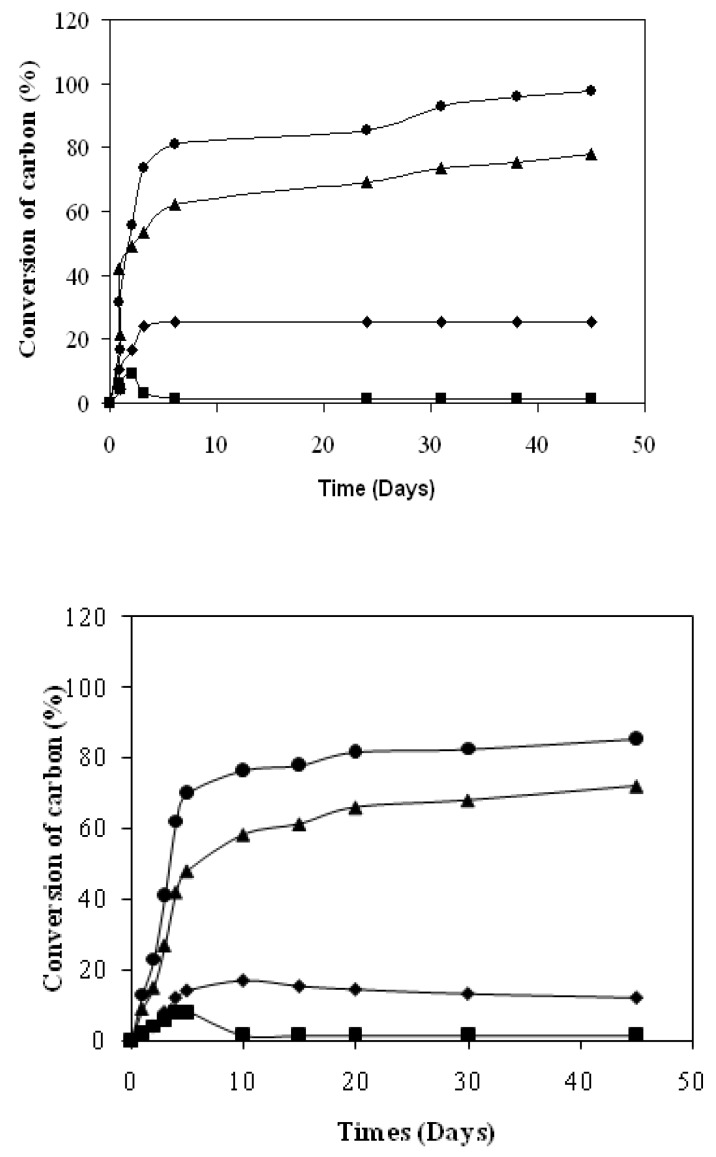
**A.** Carbon balance during the co-extruded film degradation in activated vermiculit medium according to the ISO/CEN 14855-1:2005 norm. ▲: Cg; ■: Cs; ♦: Cb: ● Cd ( from Equation 2 ). C_b_ :polymer carbon C_polymer_ is converted by micro-organisms into biomass, Cg : polymer carbon C_polymer_ is converted into carbon dioxide, Cs : polymer carbon C_polymer_ is converted into dissolved organic carbon. **B.** Carbon balance during the co-extruded film degradation in no activated vermiculit medium according to the ISO/CEN 14855 norm [16]. ▲: Cg; ■: Cs; ♦: Cb; ●: Cd ( from Equation 2 ). C_b_ :polymer carbon C_polymer_ is converted by micro-organisms into biomass, Cg : polymer carbon C_polymer_ is converted into carbon dioxide, Cs : polymer carbon C_polymer_ is converted into dissolved organic carbon.

**Figure 5:**
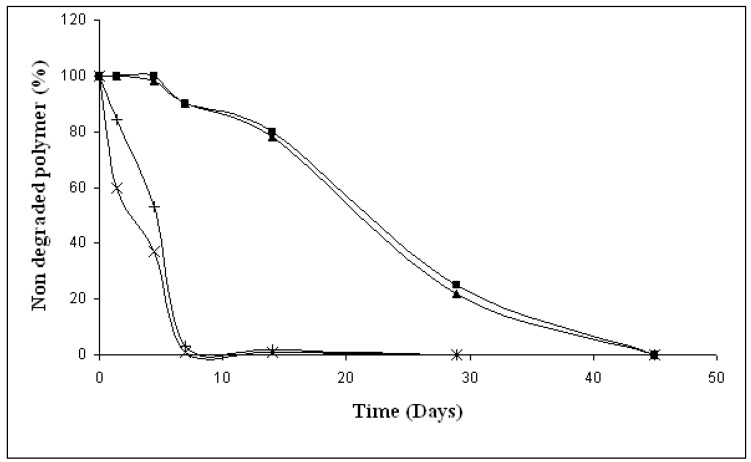
Evolution of respectively non degraded PLA part and non degraded starch part of co-extruded material during film degradation in activated vermiculite (▲), (x) and in no inactivated vermiculite (■), (+).

The mineralization curve (Cg) shown in Figure 4A shows a latency period. During the first day, the material disintegrates. So, the starch becomes reachable to the micro-organisms when the PLA remains as sheet form. Then, the starch is quickly mineralized as shown in Figure 5: by the end of the seventh day, the starch mineralization has ended and its final percentage represents 63% of the initial carbon in the material. Beyond the seventh day, the slope increase points out the mineralization of another compound, the PLA (Figure 5). Finally, at the end of the experiment, the mineralization percentage reaches 75%.

**Figure 6:**
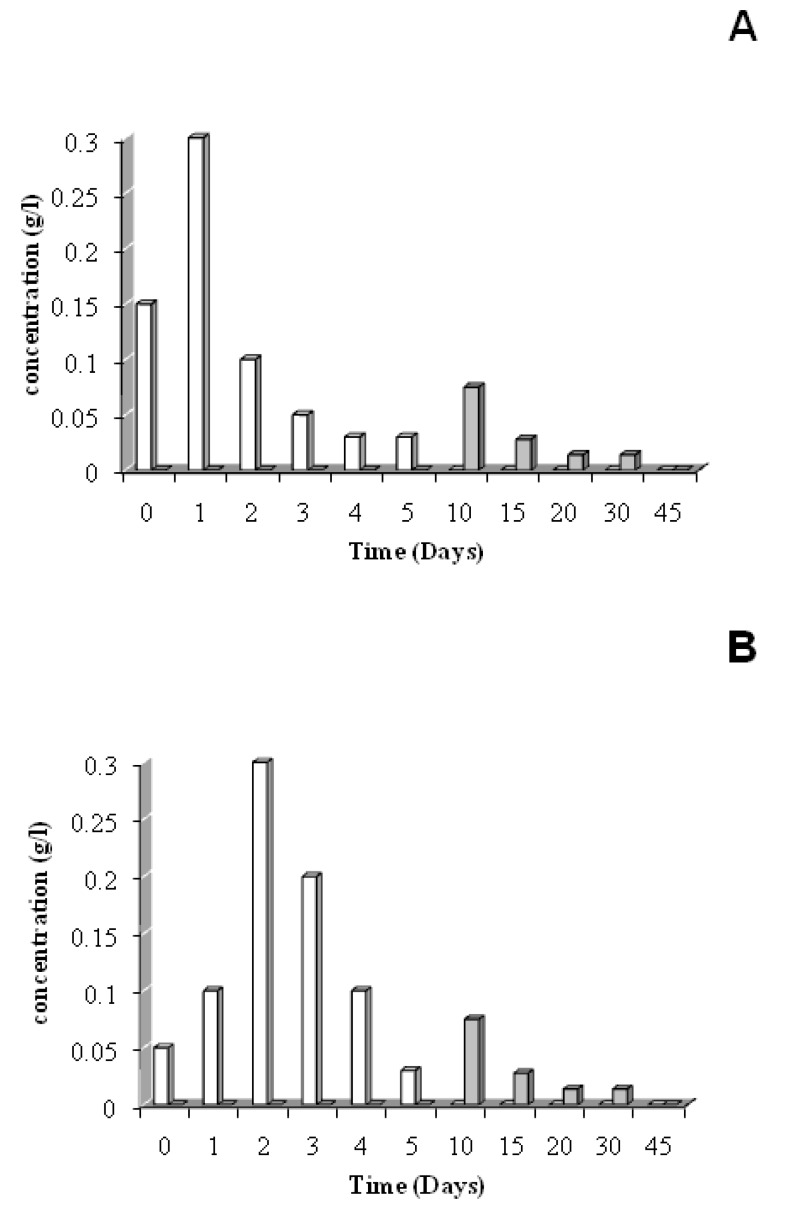
Evolution of some released soluble products (glucose (□) and acid lactic (■)) during the degradation of co-extruded material in activated inert solid medium (A) and in no activated inert solid medium (B).

In Figure 4A, the dissolved organic carbon (Cs) represents 12% of the initial material carbon after two experiment days. Then, this proportion decreases continuously: 7% the 5^th^ day and only 2% at the end of the experiment. The HPLC analysis of this dissolved fraction shows (Figure 6A) shows glucose and lactic acid as the major degradation products of the co-extruded substrate. The maximum of the released glucose quantity corresponds to the same point as the maximum of the Cs value, whereas the lactic acid appears only on the 10th day when the degradation of the starch fraction is almost achieved. This fact confirms the degradation of the two components of the material.

The biomass that is formed to the detriment of the material (Cb) increases during the two first days and represents 22% of the initial carbon. Then, this production is slowed down and only 18% of the material carbon is bioassimilated after 45 days (Figure 1). The Cd degraded carbon calculated by the Equation (2) achieved 83% of the initial carbon as early as 5 experiment days. The Cd final result (95%) compared to the Cnd residual material percentage (5%) shows thanks to the Equation (1) that all the degradation products are well identified at the end of the experiment.

#### 3.2.2. Non activated Vermiculite medium

Like activated medium, the mineralization (Cg) of the substrate in this medium shows a latent period for the same reasons (Figure 4B). Then, the starch is quickly mineralized like in the activated vermiculite medium, but the starch mineralization is ended only at the end of the 10th day and its final percentage represents 58% of the initial carbon in the material. Beyond the tenth day, the slope increase points out the mineralization of another compound, the PLA. Finally, at the end of the experiment, the mineralization percentage reaches 72%.

In this inert solid medium, we can observe a particular increase of the dissolved organic material at the day 5th versus the 2th for activated medium. The biomass that is formed to the detriment of the material (Cb) increases during the four first days against the two first days for activated vermiculite and represents 12% of the initial carbon. However, after 45 days of experiment, all the degraded carbon represents 85% of the initial substrate carbon with the non activated vermiculite.

The HPLC analysis of this hydrolyzed fraction shows (Figure 6B) glucose and lactic acid as the major degradation products of the co-extruded substrate. The maximum of the released glucose quantity happens at the same time as the maximum of the Cs value, like with activated vermiculite, but at day 2 against 1 for activated medium. We have observed that the lactic acid shows the same time lag as this observed for starch degradation.

### 3.3. Residual material studies

**Figure 7:**
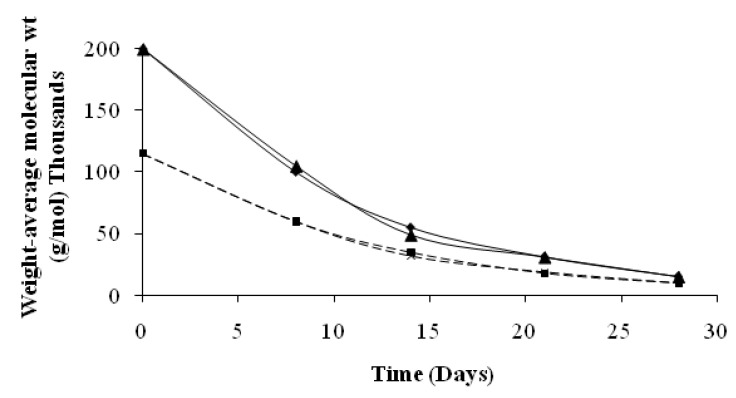
Evolution of the molecular weight of non-degraded PLA as a function of the ageing time for Mw (♦) and Mn (■) in activated inert solid medium, Mw (▲) and Mn (x) in no activated inert solid medium.

Figure 7 shows the molecular weight evolution of the non-degraded PLA. We observed a rapid diminution of both Mw and Mn at day 8 [10,11]. The values continue to decrease slowly at day 15 and remain stable in the last stages of the degradation test. The polydispersity index is 1.72 and 1.5, respectively, for the last two samples for activated and non activated medium, respectively.

This evolution is typical of the degradation mechanism of PLA which has been previously characterized in liquid medium [12] or in soil [13]. We think that random ester bond scissions due to abiotic factors induces a drastic decrease of the molecular weights. This step occurs preferentially in the amorphous region of the polymer matrix. As a result, the remaining PLA in the last stages of the test, is mainly composed of highly crystalline low molecular weight residues which are strongly resistant to biotic degradation.

During the eight first day, the mineralization rate of PLA remains very weak and starts to increase significantly following the decrease of molecular weight (Figure 7). Thus, one can deduce that around days 11 or 12, thanks to polymer abiotic hydrolysis, PLA soluble low molecular weight oligomers are released in the degradation medium from polymers matrix. Those oligomers are used by micro-organisms, leading to production of carbon dioxide and new biomass. Consequently the proportion of carbon contained in remaining PLA also decreases, as observed on Figure 5.

**Figure 8:**
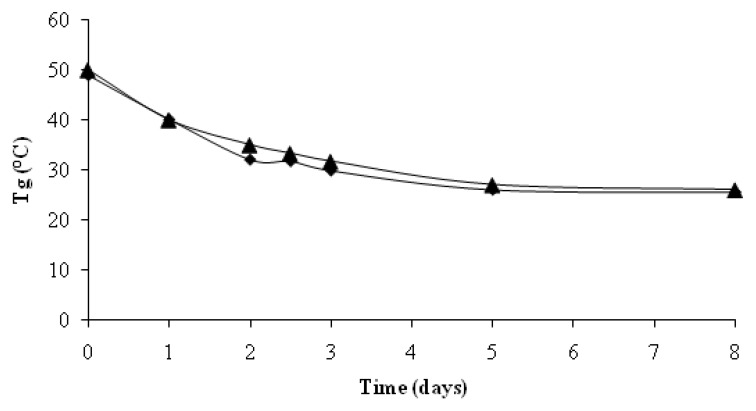
Evolution of the glass transition temperature of non-degraded PLA as a function of the ageing time in activated inert solid medium (▲) and in no activated inert solid medium (♦).

The random chain scission pointed out by SEC has also some consequences on the evolution of glass transition temperature: the Tg decreases from 50 °C (day 0) to approximately 26 °C (day 8) [13]. (Figure 8). Thus the Tg of remaining PLA is below the test temperature, meaning that the polymer is now in its rubbery state. The Tg never returns to values above the temperature of the test until the end. First, this Tg decrease enhances the water absorption in the polymer matrix and, the abiotic random chain scission reaction also. Thus, the diffusion of soluble released oligomers in the degradation medium will also be improved. Temperature is an important parameter of PLA degradation as it has an influence on the first abiotic step of its mineralisation. The physico-chemical analysis proves that the PLA has undergone some radical structural modifications due to abiotic degradation, meaning that a first abiotic step of PLA degradation has clearly occurred. Hence we think the mineralization rate should have been greater. A mature compost is a natural buffered medium thanks to the presence of various organic and inorganic compounds. Some authors have shown that no pH variation occurred during PLA degradation in compost if the proportion of PLA is below 30% by weight [14]. Our inert solid medium is also buffered by the mineral solution at pH = 7.2. Consequently, the low mineralization rate was not due to a limitation of micro-organisms mineralization activity caused by a pH decrease.

## 4. Conclusions

With regard to biodegradation in different media, the final mineralization percentages for the co-extruded material in inert solid activated, no activated and composting media were 75%, 72% and 76%, respectively. These values allowed us to conclude that these co-extruded materials may be considered to be biodegradable, whatever the medium. The speed of biodegradation mainly depended on the nature of the medium and on the method of activation that was used.

As we have showed in our work, the use of vermiculite as an inert solid support can predict how co-extruded starch/poly(lactic acid) polymeric material can degrade when it would be stored in compost. Vermiculite medium can simulate the mechanism of biodegradation of a polymer.

It is very important that the activation dose must contain a carbon source with a similar structure that the one of the polymer tested. It should allow the growth and the presence of a numerous and highly active microbial flora when co-extruded starch/poly(lactic acid) polymeric material carbon is disposable for micro-organisms. Thus one can obtain both a greater mineralization rate and a better simulation of co-extruded starch/poly(lactic acid) polymeric material degradation in compost.

In our work, the activated dose contains starch and cellulose as carbon source. As shown by [15] the use of this activated dose improves the biodegradation of starch in an inert solid support to predict compost degradation. However, we show that we do not obtain the same effect on the PLA part of co-extruded starch/poly(lactic acid) polymeric material.

Activated dose shows no action in the biodegradation of the PLA part. However, the final percentage of biodegradation is the same for each medium, but the kinetics of biodegradation of co-extruded starch/poly(lactic acid) polymeric material appears slower for non activated inert medium. The physico-chemical analysis (DSC and SEC) of PLA part during tests show that dose of activation has no additional effect on its physico-chemical characteristics.

In conclusion, the choice of the composition of the activation dose is a fundamental step to predict biodegradation of a polymer stored in compost and depends on kind of polymer tested.
